# Assessment of autostereoscopic perception using artificial intelligence-enhanced face tracking technology

**DOI:** 10.1371/journal.pone.0312153

**Published:** 2024-10-17

**Authors:** Bo Yu, Lu Liu, Ning Yang, Lingzhi Zhao, Huang Wu

**Affiliations:** Department of Optometry, The Second Hospital of Jilin University, Changchun, China; Hadassah Academic College, ISRAEL

## Abstract

**Purpose:**

Stereopsis, the ability of humans to perceive depth through distinct visual stimuli in each eye, is foundational to autostereoscopic technology in computing. However, ensuring stable head position during assessments has been challenging. This study evaluated the utility of artificial intelligence (AI)-enhanced face tracking technology in overcoming this challenge by ensuring that each eye consistently receives its intended image.

**Methods:**

The Lume Pad 2, an autostereoscopic tablet with AI-enhanced face tracking, was utilized to simulate quantitative parts of the Stereo Fly test and TNO Stereotests for contour and random dot stereopsis. The study recruited 30 children (14 males and 16 females, mean age of 9.2 ± 0.3 years, age range of 6–12 years) and 30 adults (10 males and 20 females, mean age of 29.4 ± 1.0 years, age range of 21–42 years) to assess the tablet’s inter-session reliability. Agreement between conventional and the autostereoscopic tablet-simulated stereotests was tested in a larger group of 181 children (91 males and 90 females, mean age of 9.1 ± 0.4 years, age range of 6–12 years) and 160 adults (69 males and 91 females, mean age of 38.6 ± 2.1 years, age range of 21–65 years). Inter-session reliability and agreement were analyzed using weighted Kappa coefficient and non-parametric Bland-Altman analysis.

**Results:**

The autostereoscopic tablet demonstrated high inter-session reliability (*κ* all > 0.80), except for the simulated TNO Stereotest in adults, which demonstrated moderate inter-session reliability (*κ* = 0.571). Non-parametric Bland-Altman analysis revealed zero median differences, confirming consistent inter-session reliability. Similar patterns were observed in comparing AI-based and conventional methods, with both the weighted Kappa coefficient (*κ* all > 0.80) and non-parametric Bland-Altman analysis indicating significant agreement. The agreement between methodologies was confirmed by permissible differences, which were smaller than the minimum step range.

**Conclusion:**

The integration of AI-based autostereoscopic technology with sub-pixel precision demonstrates significant potential for clinical stereopsis measurements.

## Introduction

Stereopsis represents the capability to discern depth due to distinct visual stimuli from each of the two eyes [1]. This depth perception originates from the separation of our eyes, which allows them to perceive slightly varied angles of a given scene [2]. These variations, termed binocular disparity, are integrated by the brain to produce a three-dimensional (3D) perception [3].

Assessing stereopsis is vital in diagnosing and overseeing specific ocular conditions, particularly during early developmental stages. This assessment can signal conditions such as amblyopia or strabismus [4–7]. Primarily, two categories exist for stereopsis evaluation: real space and dichoptic presentation. In the real space approach, both eyes observe an identical object. However, the inherent interpupillary distance causes each eye to receive a unique image. The brain amalgamates these variances, leading to the formation of stereopsis. Several instruments enable real space stereopsis evaluation, such as the Howard-Dolman apparatus [8, 9] and the Frisby Near Stereotest [10–12]. Conversely, in the dichoptic presentation, a distinct method is employed to differentiate the visual stimuli for each eye. Under the condition of dichoptic presentation, the visual stimulus intended for the right eye is invisible to the left eye, and vice versa [13]. Typically, specialized eyewear, such as the anaglyphic glasses in the TNO Stereotest [14–17] or the polarized glasses in the Titmus Fly Stereotest [17, 18] and Randot Stereotest [12, 19], serve this purpose.

A specialized evaluation method, known as the autostereoscopic technique, employs dichoptic presentation to enable the eyes to directly observe each eye’s object without the need for auxiliary equipment. This technique is applied in the Lang Stereotest [20, 21]. This test utilized lenticular technology which includes a plastic overlay with a smooth side and another side with a series of aligned, thin convex lenses which is the lenticular side. Positioned beneath this sheet is an interlaced image, often derived from multiple captures at different angles, tailored to produce 3D effects [22].

The autostereoscopic technic often facilitates better cooperation, especially among younger subjects. However, the technology has inherent limitations. Notably, while the lenticular lens can effectively ensure that each eye perceives a unique image, it does not allow for the determination of which specific image is received by each eye. Moreover, while the autostereoscopic technique can establish disparity in test pages to evoke stereopsis, it remains undeterminable whether the observed disparity is crossed or uncrossed. For instance, during the Lang Stereotest, individuals with adept stereopsis might notice that slight head movements or minor vertical adjustments to the test plate can shift the perceived depth of the stereopsis symbols. The symbols might appear to emerge from the plane in one angle (crossed disparity) and recede into it at another (uncrossed disparity). Those with compromised stereopsis may observe the symbols oscillating horizontally with any slight positional shift, potentially leading to the identification of preset symbols and consequent false positives [23]. Hence, maintaining a stationary head and test plate position, especially among children, poses a considerable challenge during evaluations. In addition, due to the lenticular lenses distributing the display’s resolution between both eyes, each eye usually perceives only half of the original image’s horizontal resolution, potentially reducing perceived image clarity. Since stereopsis tests depend on precise depth cues to assess an individual’s depth perception, any minor misalignment or manufacturing flaw in the lenticular lens might produce inaccurate results [24].

**Fig 1:**
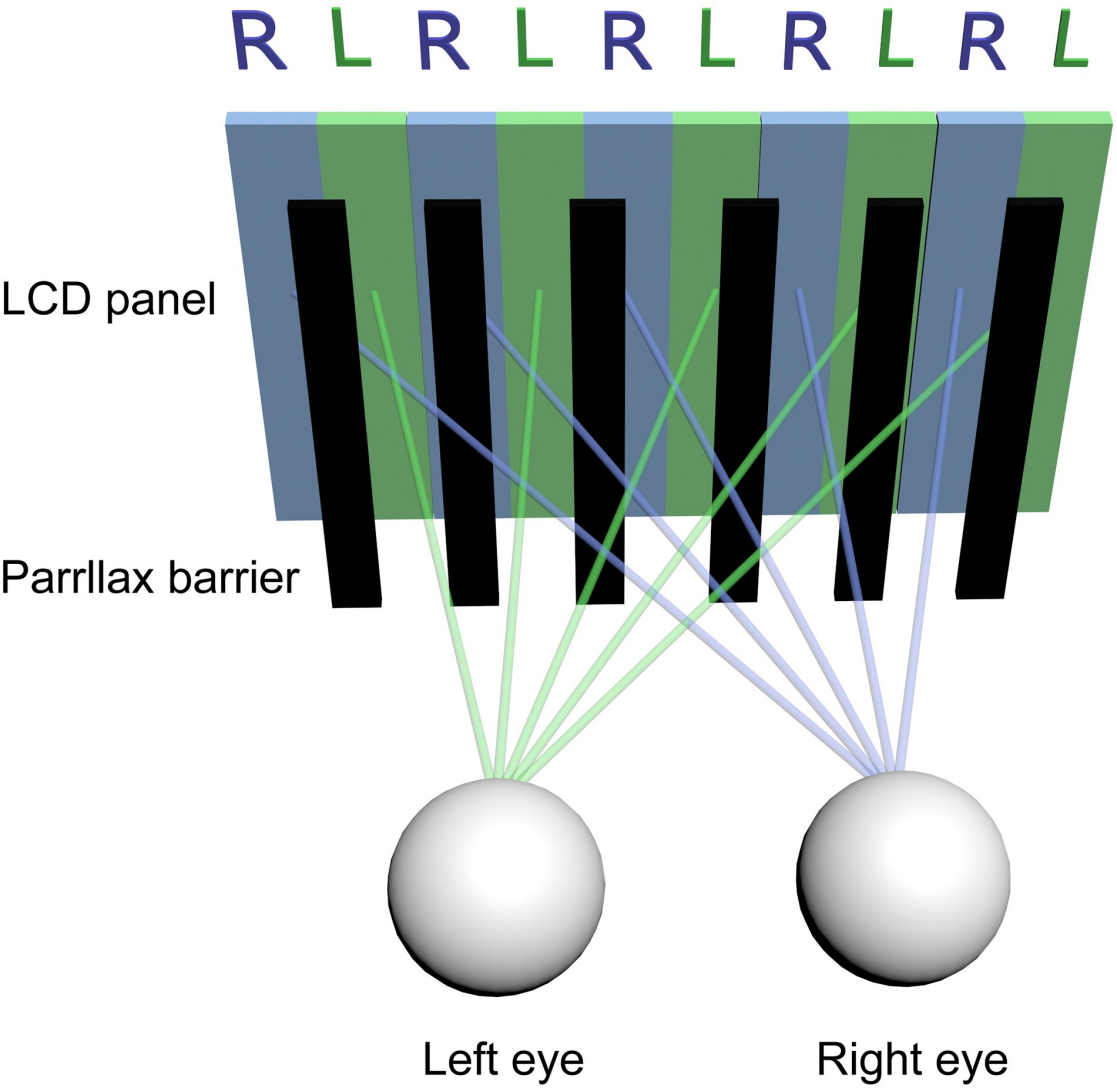
Diagram for parallax barrier technology. Two interlaced images are simultaneously displayed on the LCD panel. Positioned in front of the panel, the parallax barrier directs the green light to the left eye and the blue light to the right eye, thereby producing an impression of stereopsis.

For years, autostereoscopic technology has been integrated into computer systems. Within this domain, the parallax barrier technology is utilized to generate autostereoscopic displays [25–27]. This technique modulates the angle of light emission from the display, ensuring distinct images reach each eye, thereby producing an impression of stereopsis (Fig 1). The parallax barrier is a precision-layered component positioned in front of the display. This component features meticulously aligned slits. Behind this barrier, two interlaced images are displayed concurrently, each tailored for a specific eye. The barrier’s slits permit each eye to view only its corresponding image, with one image’s light directed to one eye and the second image’s light to the other, resulting in the 3D perception. For optimal 3D visualization, the observer must be situated within a particular range, commonly termed the "sweet spot." Beyond this zone, the 3D experience may diminish or become absent.

Our previous study utilized autostereoscopic smartphones to simulate the Lang Stereotest I & II, Pass Test 3, Dinosaur Stereoacuity Test, and the Random Dot Stereo Acuity Test. The results showed that autostereoscopic smartphones are effective in stereopsis evaluation [28]. However, maintaining a stable head position to obtain the optimal 3D perception presents a challenge. Recent advances in artificial intelligence (AI) and the refinement of face tracking technology may mitigate existing concerns by accurately determining eye position. By detecting and tracking the observer’s eye positions in real-time and dynamically adjusting the output light direction of interlaced images, AI technology ensures that each eye receives the correct image, even when the observer moves their head or eyes [29]. This may achieve a viewing experience similar to anaglyphic or polarized glasses.

Conversely, autostereoscopic technology inherently halves the horizontal resolution. For instance, with a computer’s pixel pitch of 0.1mm, using regular pixels at a distance of 40cm will yield a disparity of approximately 100 seconds of arc (″), spanning 2 pixels. While this precision aligns with the Lang stereotest standard, it falls short of the requirements for more accurate conventional stereotests. However, the implementation of sub-pixel technology offers a solution. Essentially, sub-pixel rendering (SPR) technology enhances display resolution by manipulating the image to allow displacements that are on a scale smaller than one pixel. This facilitates precise control over the disparity settings used in stereotests, exceeding the native resolution of the display. That is, for the computer mentioned above, a disparity of 10″ (spanning 0.2 pixels) at 40cm can be achieved using SPR.

This study is the first to investigate whether the dichoptic presentation achieved using AI-enhanced autostereoscopic technology is comparable to that obtained with anaglyphic or polarized glasses, offering a novel perspective on stereotest methods. Through SPR technology, we simulated the quantitative sections of the Stereo Fly Test (ranging from 40″ to 800″) for contour stereopsis evaluation, and the TNO Stereotest (spanning 60″ to 480″) for random-dot stereopsis evaluation. Inter-session reliability and agreement analysis were then applied to investigate the reliability of this approach.

## Materials and methods

### Participants

This prospective and comparative study was conducted at the Second Hospital of Jilin University from August 1, 2023 to January 10, 2024. A total of 211 primary students and 190 adult participants were recruited for this study. Of these, 30 primary students and 30 adults were included in the inter-session reliability analysis. A different group of 181 primary students and 160 adults participated in the agreement analysis. All participants met the following inclusion criteria: (1) Ages 6 to 12 years for the children’s group and over 18 years for the adult group; (2) Best-corrected visual acuity of 0 logMAR or better; (3) Refractive error not exceeding ±3D sphere and ±1D cylinder; (4) Anisometropia not excessing 1D; (5) Voluntary participation and capability of cooperating with the examination. Participants with strabismus, amblyopia, dry eye disease, retinal diseases, systemic diseases, or previous surgery for eye disease were excluded. Participants did not receive financial compensation for their participation. The study design adhered to the Declaration of Helsinki and was approved by the Ethics Committee of the Second Hospital of Jilin University (Approval No. 2020–110 for adults and 2020–111 for children). Before participation, written informed consent was obtained from each adult participant and the guardians of all underage participants, and verbal consent was also obtained from each underage participant.

### Equipment and test symbols

The autostereoscopic tablet employed the Lume Pad 2 (Leia Inc., Menlo Park, CA, USA). This device features a display size of 12.4 inches and a resolution of 2560×1600 pixels. When assessed at a distance of 0.36m, a disparity of 1 pixel corresponded to 60″, whereas at 0.43m, it corresponded to 50″. Due to the characteristics of the autostereoscopic display, the minimum achievable disparity was 2 pixels, corresponding to a whole pixel presentation disparity of 120″ at 0.36m or 100″ at 0.43m. To achieve finer disparities, we employed SPR technology. All test symbols were produced using a program developed in C# and integrated with the OpenCV 3.0 development kit. The bilinear interpolation algorithm facilitated sub-pixel shifts of these test symbols.

**Fig 2:**
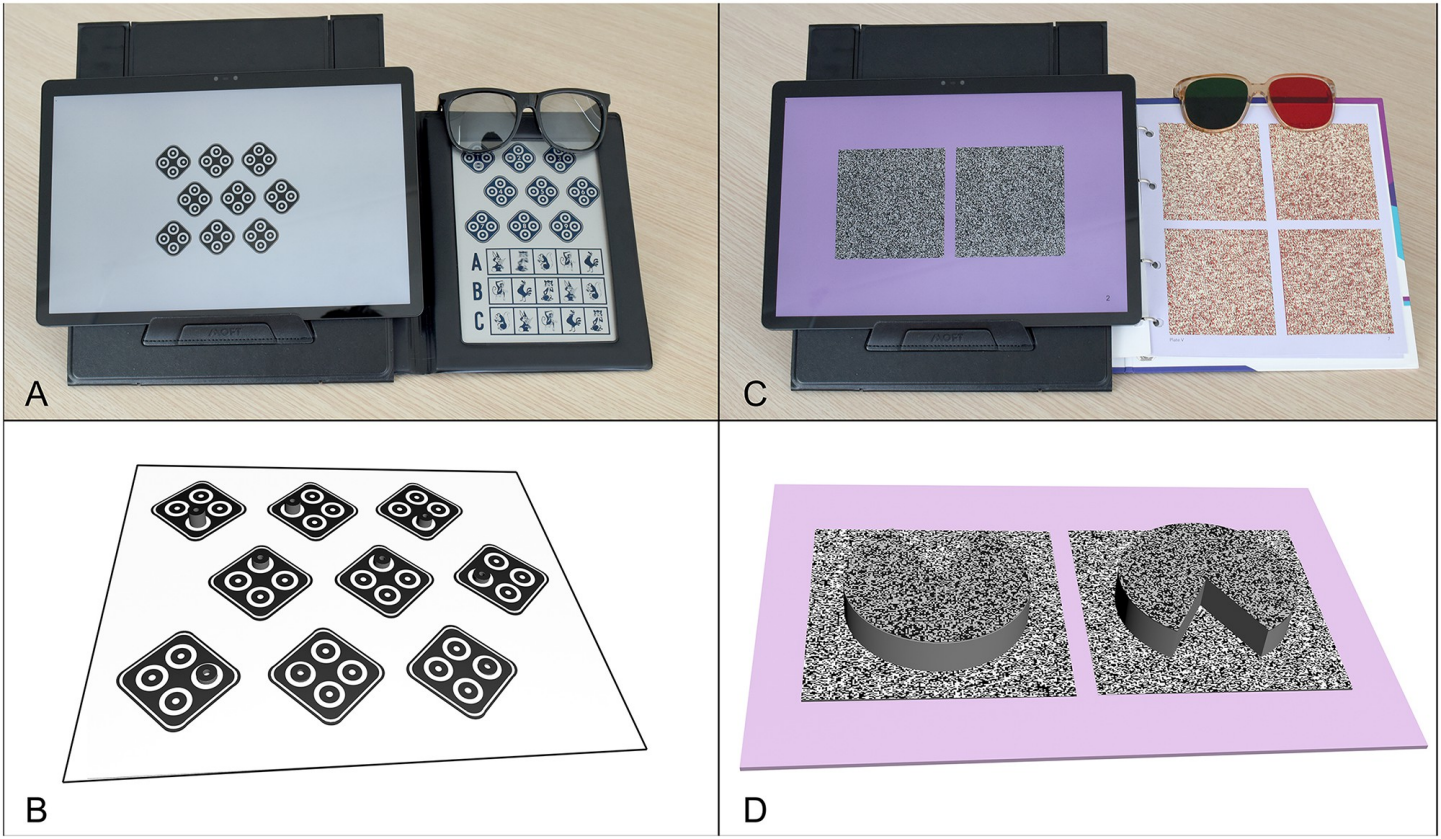
The diagrams of the stereotest and the simulated perceptions from the test images. (A) Illustrates the Stereo Fly test alongside its simulation on the AI-enhanced autostereoscopic tablet. (B) This section simulated the perceptions when a participant’s stereothreshold was lower than the disparity shown. In such instances, the target appeared to protrude. The orientation of the stereo symbol varies: In the first row, it is positioned at the bottom with a disparity of 800″, to the left with 400″, and again at the bottom with 200″. The second row places the stereo symbols at the top with disparities of 140″ and 100″, and then to the left with 80″. The third row showcases the stereo symbols on the right with a disparity of 60″, to the left at 50″, and once again on the right at 40″. (C) Displays the TNO Stereotest alongside its simulation on the AI-enhanced autostereoscopic tablet. (D) Spotlights a 240″ test pattern. For participants whose stereothreshold is below 240″, their perception of the first Pacman would have its mouth oriented downwards, while the second Pacman’s mouth would be facing upwards.

The contour-based stereo symbol simulated the quantitative measurement segment of the Stereo Fly Test (Stereo Optical Company, Inc., in Illinois, USA). This symbol, represented as a diamond, is adorned with four equidistant black rings placed at its corners. One of the circles is designed for crossed disparity. Disparity values set for this symbol included 800″ (2.9 log arcsec), 400″ (2.6 log arcsec), 200″ (2.3 log arcsec), 140″ (2.1 log arcsec), 100″ (2.0 log arcsec), 80″ (1.9 log arcsec), 60″ (1.8 log arcsec), 50″ (1.7 log arcsec), and 40″ (1.6 log arcsec). The largest disparity of 800″ indicated reduced stereopsis, whereas the smallest disparity of 40″ corresponded to normal stereopsis. Refer to Fig 2 for the evaluation and simulation diagrams.

For the random-based stereo symbol, our design simulated the quantitative measurement section of the TNO Stereotest (Lameris Ootech BV, Ede, Netherlands). This symbol depicted a "Pacman" with its "mouth" oriented upwards, downwards, to the left, or to the right. The disparity settings for this symbol were 480″ (2.7 log arcsec), 240″ (2.4 log arcsec), 120″ (2.1 log arcsec), and 60″ (1.8 log arcsec) (Fig 2).

### Stereopsis measurement

#### Test distance

The conventional stereotest distance was set at 0.4m. For the TNO Stereotest simulation on the AI-based autostereoscopic tablet, the distance was adjusted to 0.36m, whereas for the Stereo Fly Test, it was increased to 0.43m. These adjustments ensured that the disparity settings were aligned with the conventional stereotests and optimized pixel utilization. The autostereoscopic tablet remained stationary throughout the stereopsis evaluation period. Before the test, the eye-to-tablet distance was determined by instructing each participant to hold a tape measure from their eye to the tablet, which was placed on a table. Participants were instructed to avoid moving their heads. After the test was completed, the examiner asked participants if they perceived any alternations between the protrusion and indentation of the stereo symbols.

#### Conventional and autostereoscopic tablet simulations of the Stereo Fly Test

Polarized glasses were required for the conventional Stereo Fly Test. The evaluation began with a maximum disparity of 800″. Participants, both children and adults, were tasked with looking at the four circles and reporting which circle appeared closer to them or protruded from the tablet. If participants failed to complete the Stereo Fly Test successfully, a score of 1600″ was recorded. Upon correct identification of the circle, the disparity was reduced to 400″, and testing continued until the participant could no longer identify the circle accurately. The last correctly identified disparity was recorded as the subject’s stereoacuity.

#### Conventional and autostereoscopic tablet simulations of the TNO stereotest

Anaglyphic glasses were mandatory for the conventional TNO stereotest. The evaluation started with a disparity of 480″. Participants were tasked with indicating the orientation of Pacman’s mouth: up, down, left, or right. If participants were unable to successfully complete the TNO stereotest, a score of 960″ was recorded. If the participant chose correctly, the disparity decreased to 240″ and continued to decrease until a wrong choice occurred. The last correct disparity was recorded as the participant’s stereoacuity.

Both 1600″ and 960″ represent the next log level (0.3 log arcsec increment) above the largest disparity of the Stereo Fly Test and TNO stereotest, respectively. Assigning the next log level to failed tests is a common practice in stereoacuity data analysis [10], enabling calculations of differences between tests and test-retest reliability.

### Test procedure

All participants were examined by one ophthalmologist. Each participant’s age and gender were recorded, and refraction, visual acuity, slit-lamp biomicroscopy, and fundoscopy were examined. Participants with refractive error or presbyopia wore their appropriate refractive correction lenses for testing if needed. All stereotests were conducted under standard illumination in the same quiet, soundproof room. During the test, the subject remained seated with the test page perpendicular to their gaze direction.

#### Inter-session reliability and agreement analysis

**Fig 3:**
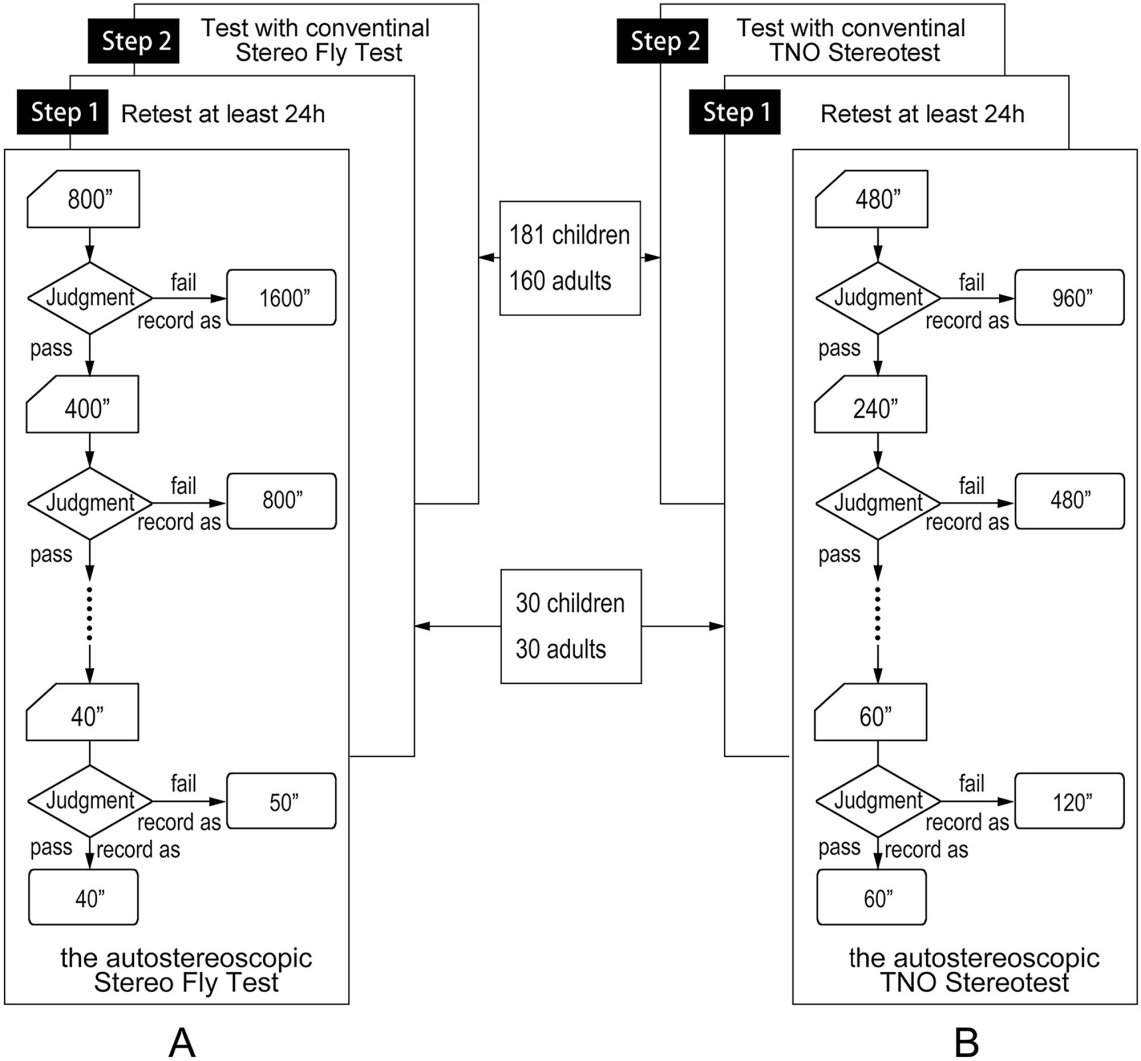
Flow chart of the test procedure. The study comprised two tests: (A) the autostereoscopic Stereo Fly Test and (B) the autostereoscopic TNO Stereotest. For each participant, the order of these two tests was randomly assigned. Step 1 involved repeated measurements of participants’ stereoacuity with a mandatory minimum interval of 24 hours to assess the inter-session reliability of the autostereoscopic stereotests. Step 2 involved participants randomly completing both the autostereoscopic and conventional Stereo Fly Test and TNO Stereotest to evaluate the agreement between the autostereoscopic and conventional stereotests.

For the inter-session reliability analysis, each participant underwent an autostereoscopic tablet-simulated stereotest twice, with a mandatory minimum interval of 24 hours between the two evaluations to reduce the influence of learning effects (Fig 3). To determine whether each eye received the correct image, participants were instructed to view the test page with the largest disparity when covering the left eye, the right eye, and with both eyes open before the first test began. Participants were tasked with identifying under which conditions the stereo symbol could be observed and whether it appeared protruded or indented on the screen.

The quantitative measurements from both the conventional Stereo Fly Test and the TNO Stereotest, alongside their counterparts on the autostereoscopic tablet, were utilized for agreement analysis. In total, four tests were administered: the conventional Stereo Fly Test, the conventional TNO Stereotest, the autostereoscopic Stereo Fly Test, and the autostereoscopic TNO Stereotest. The sequence of these tests was randomized (Fig 3).

### Statistical analysis

The stereoacuity, treated as an ordinal categorical variable, was transformed from seconds of arc to log arcsec for analysis. All data were processed and analyzed using SPSS (version 26.0; IBM Corp, Armonk, NY, USA) and MedCalc (version 22.023; MedCalc Software bvba, Ostend, Belgium). The Shapiro-Wilk test was employed to assess the normality of data distributions. For normally distributed data, a paired t-test was used to analyze differences between stereotests, while inter-session reliability of the autostereoscopic simulated stereotests and their agreement with conventional stereotests were evaluated using Bland-Altman analysis and the Intraclass Correlation Coefficient (ICC). For non-normally distributed data, the Wilcoxon signed-rank test, non-parametric Bland-Altman analysis, and the weighted Kappa coefficient were employed to evaluate the reliability and agreement. The permissible difference for non-parametric Bland-Altman analysis was defined by the interval extending from the lower to the upper bounds of the 95% confidence intervals (CI) for the limits of agreement (LoA). The weighted Kappa coefficient categorized agreement strength using the kappa (κ) statistic: κ<0 indicated extremely poor agreement; 0.01≤κ≤0.20, slight agreement; 0.21≤κ≤0.40, fair agreement; 0.41≤κ≤0.60, moderate agreement; 0.61≤κ≤0.80, substantial agreement; 0.81≤κ≤1.0, almost perfect agreement [30]. A p-value of less than 0.05 was considered statistically significant.

## Results

The demographics of the participants are shown in Table 1. All participants failed to observe the stereo symbol under monocular viewing conditions. None of the participants reported any alternation between protrusion (crossed disparity) and indentation (uncrossed disparity) in the stereo symbols.

**Table 1:** Demographics of the participants.

	Inter-session reliability analysis		Agreement analysis	
	Children (*n* = 30)	Adult (*n* = 30)	Children (*n* = 181)	Adult (*n* = 160)
Age (Mean±SD, years)	9.2 ± 0.3	29.4 ± 1.0	9.1 ± 0.4	38.6 ± 2.1
Age range (years)	[6, 12]	[21, 42]	[6, 12]	[21, 65]
Gender, n (%)				
Males	14 (46.7%)	10 (33.3%)	91 (50.3%)	69 (43.1%)
Females	16 (53.3%)	20 (66.7%)	90 (49.7%)	91 (56.9%)

### Inter-session reliability of the stereotest simulated on AI-based autostereoscopic tablet

Test 1 and Test 2 of the AI-based autostereoscopic Stereo Fly Test denote two evaluations using contour-based symbols. Similarly, Test 1 and Test 2 of the AI-based autostereoscopic TNO Stereotest correspond to two evaluations involving random-based symbols.

**Fig 4:**
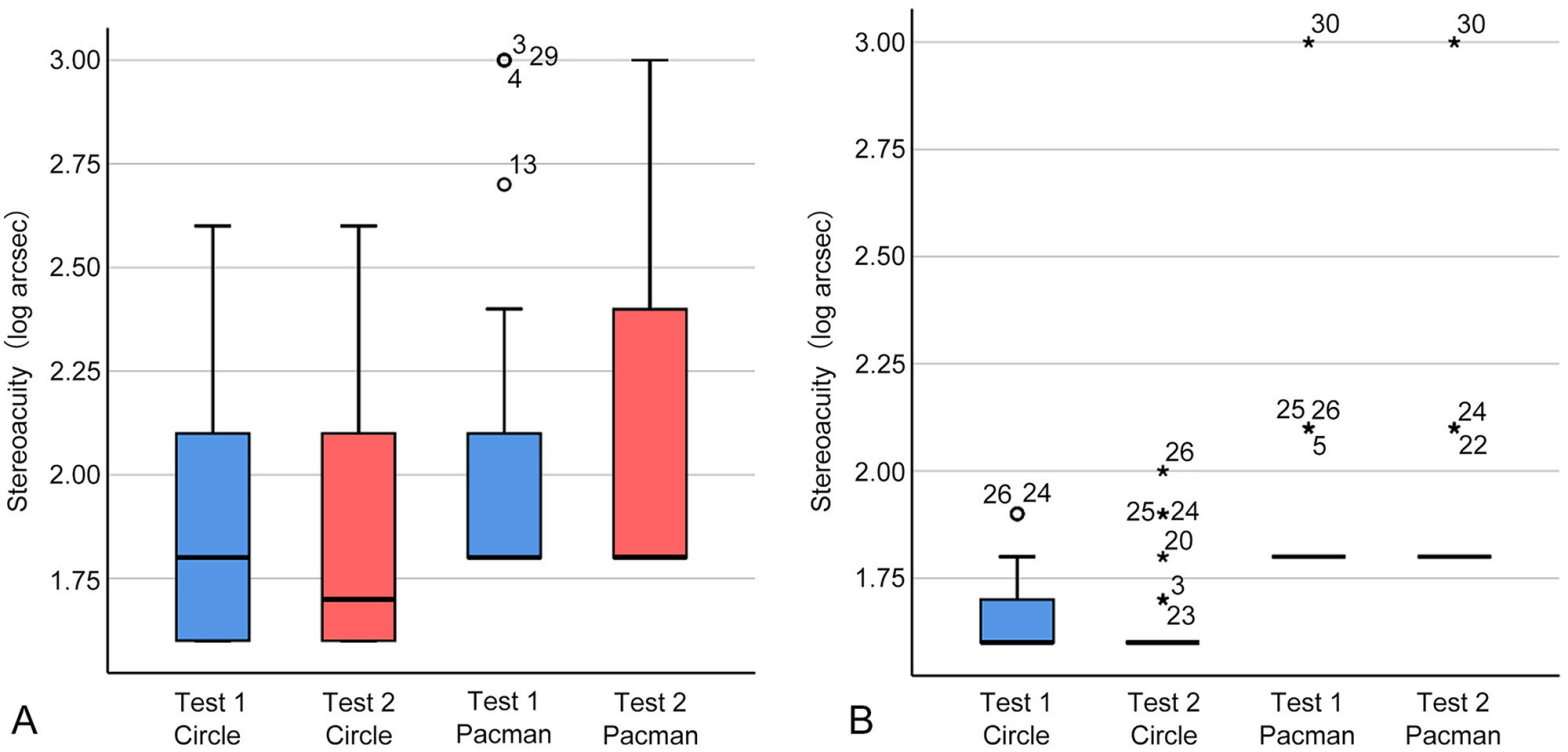
Boxplots for both children and adults. Boxplots for children (A) and adult (B) subjects. The box represents the interquartile range (IQR), with the bottom representing the first quartile (Q1, 25th percentile), and the top representing the third quartile (Q3, 75th percentile). The line within the box represents the median or central data point. Whiskers extending from the top and bottom of the boxes represent variability beyond quartiles typically spanning up to 1.5 times the IQR. Outliers are shown as outlying circles or asterisks, with their subject ID specified by the data symbols. Outliers below Q1 minus 1.5 times the IQR or above Q3 plus 1.5 times the IQR are marked with circles. Those exceeding 3 times the IQR are indicated by asterisks. Children: *n* = 30 participants; Adults: *n* = 30 participants.

The distribution of data is illustrated in the boxplot in Fig 4, and the statistical results are detailed in Table 2. Notably, for the adult group, the interquartile range for Test 2 of the autostereoscopic Stereo Fly Test and for both Test 1 and Test 2 of the autostereoscopic TNO Stereotest was 0.00 to 0.00. Specifically, the stereoacuity at the 2.5th percentile, median, and 97.5th percentile for these tests was identical, resulting in the boxplots collapsing into a straight line at the median. The Shapiro-Wilk test revealed a significant deviation from the normality of all groups (*P* all < 0.001). Therefore, the Wilcoxon Signed-Rank test was applied to evaluate the differences between Test 1 and Test 2, which did not yield any statistically significant differences (*P* all > 0.05). Furthermore, the weighted Kappa coefficient indicated that, except for the autostereoscopic TNO Stereotest for adults, which demonstrated moderate inter-session reliability (*κ* = 0.571), substantial inter-session reliability was observed across all other groups (*κ* all > 0.80).

**Table 2:** Statistical results of the data.

Group	Autostereoscopic Stereotest	Test	Shapiro-Wilk test		Median (Inter-quartile range) log arcsec	Wilcoxon Signed-rank test		weighted Kappa coefficient	
			Statistic	*P*		Statistic	*P*	*κ*	*95% CI*
Child	The Stereo Fly Test	Test 1	0.735	<0.001	1.78 (1.60–2.18)	−1.127	0.260	0.962	[0.928, 0.996]
		Test 2	0.736	<0.001	1.70 (1.60–2.18)				
	The TNO Stereotest	Test 1	0.691	<0.001	1.78 (1.78–2.08)	−0.447	0.655	0.951	[0.898, 1.000]
		Test 2	0.700	<0.001	1.78 (1.78–2.38)				
Adult	The Stereo Fly Test	Test 1	0.610	<0.001	1.60 (1.60–1.70)	−1.134	0.257	0.889	[0.748, 1.000]
		Test 2	0.499	<0.001	1.60 (1.60–1.60)				
	The TNO Stereotest	Test 1	0.341	<0.001	1.78 (1.78–1.78)	−0.447	0.655	0.571	[0.016, 1.000]
		Test 2	0.293	<0.001	1.78 (1.78–1.78)				

**Fig 5:**
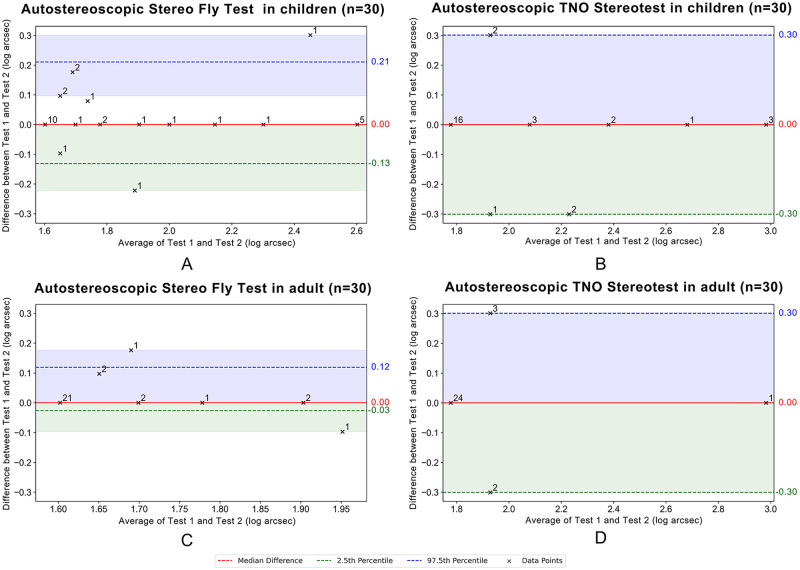
Non-parametric Bland-Altman plots between Test 1 and Test 2 of autostereoscopic stereotests in both children and adults. The y-axis represents the differences in stereoacuity measurements between Test 1 and Test 2 from AI-based autostereoscopic 3D simulated stereotests, and the x-axis represents their average, both expressed in log arcsec. Stereoacuity thresholds measured from Test 1 and Test 2 of the autostereoscopic Stereo Fly test were compared in children (A) and adults (C), and those from the autostereoscopic TNO Stereotest were compared in children (B) and adults (D). The black crosses depict the differences between the two evaluations as they relate to their average, with adjacent numbers indicating the number of participants sharing the same differences. The median difference between the measurements is indicated by the red dashed line. The green dashed line signifies the 2.5th percentile of the differences, marking the lower LoA. The blue dashed line represents the 97.5th percentile, which is the upper LoA. The accompanying shaded green and blue regions represent the 95% confidence intervals for the 2.5th and 97.5th percentiles, respectively. The 95% confidence intervals were calculated using the bootstrap method, which is suitable for data without requiring assumptions about the underlying distribution. Children: *n* = 30 participants; Adults: *n* = 30 participants.

Fig 5 shows the non-parametric Bland-Altman plot. The median difference between Test 1 and Test 2 of the autostereoscopic Stereo Fly Test among children was zero, suggesting no median bias between the two tests. The 2.5th percentile was −0.13 log arcsec and the 97.5th percentile was 0.21 log arcsec. The 95% CI for the LoA ranged from −0.22 to 0.00 log arcsec for the lower bound, and from 0.10 to 0.30 log arcsec for the upper bound at the 97.5th percentile. Consequently, the range of LoA between the 2.5th and 97.5th percentiles was determined to be from −0.13 to 0.21 log arcsec, cumulating to a total range of 0.34 log arcsec, which is approximately equivalent to 2.2″. The permissible difference between these two tests was established to be from −0.22 to 0.30 log arcsec. This constitutes a total permissible difference of 0.52 log arcsec, approximately equivalent to 3.3″ (See Table 3).

**Table 3:** Non-parametric Bland-Altman plot for inter-session reliability between Test 1 and Test 2.

Index	Children		Adults	
	Simulated Stereo Fly test	Simulated TNO Stereotest	Simulated Stereo Fly test	Simulated TNO Stereotest
Median difference	0	0	0	0
2.5th percentile (95% CI)	−0.13 (−0.22, 0.00)	−0.30 (−0.30, 0.00)	−0.03 (−0.10, 0.00)	−0.30 (−0.30, 0.00)
97.5th percentile (95% CI)	0.21 (0.10, 0.30)	0.30 (0.00, 0.30)	0.12 (0.00, 0.18)	0.30 (0.00, 0.30)
LoA	0.34	0.60	0.15	0.60
permissible difference	0.52	0.60	0.27	0.60

Simulated Stereo Fly test: the autostereoscopic Stereo Fly Test; Simulated TNO Stereotest: the autostereoscopic TNO Stereotest. All units of the data are log arcsec.

Other detailed results of Test 1 and Test 2 for the autostereoscopic TNO Stereotest for children, as well as Test 1 and Test 2 for both the autostereoscopic Stereo Fly and TNO Stereotest for adults, are shown in Table 3.

### Agreement between conventional and autostereoscopic stereotest

Group "Circle" represents the Stereo Fly Test’s quantitative segment using circles as stereo symbols, and the "Circle-AI" group represents the autostereoscopic Stereo Fly Test. Group "Pacman" signifies the TNO Stereotest’s quantitative section with Pacman stereo symbols, and the "Pacman-AI" group represents the autostereoscopic TNO Stereotest.

**Fig 6:**
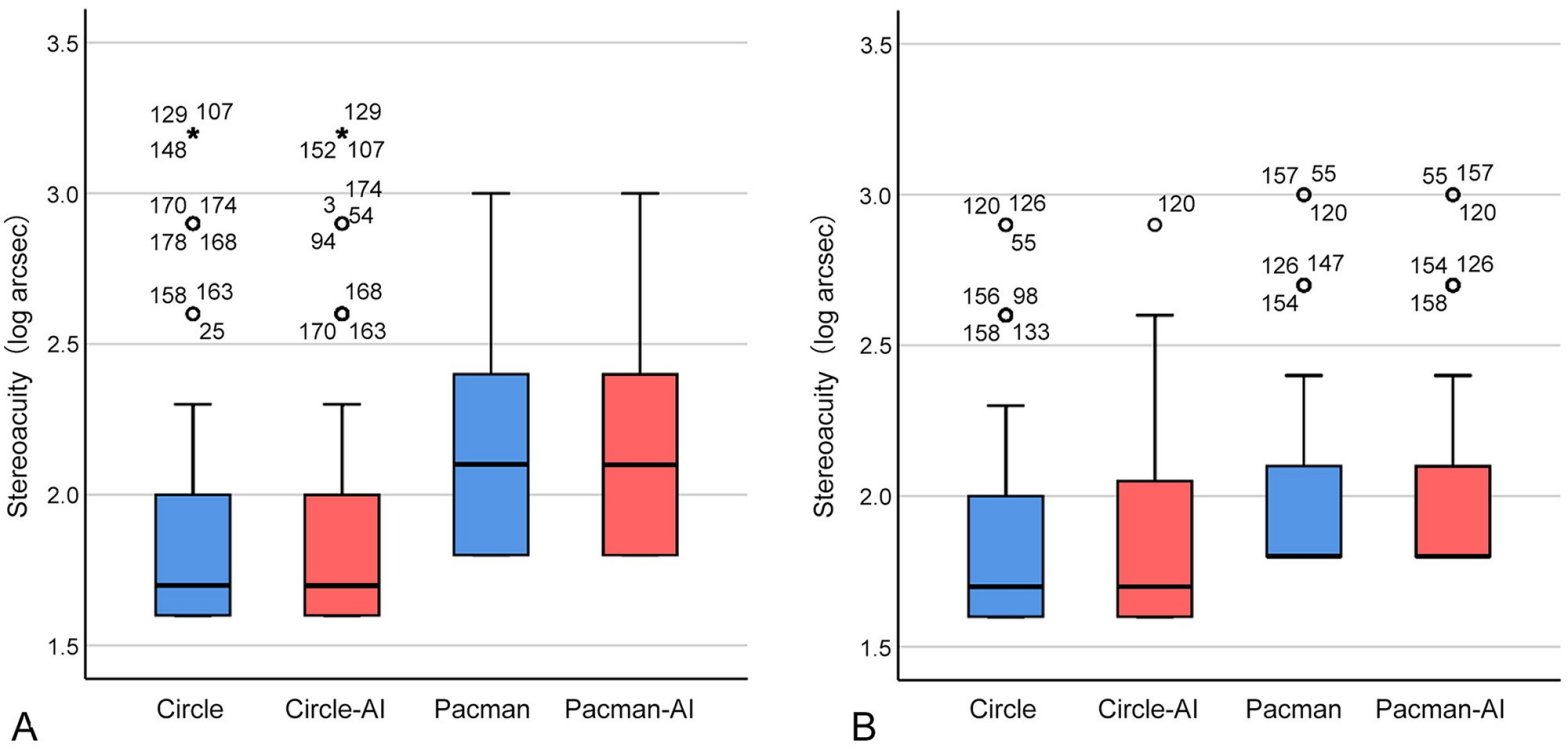
Boxplots for both children and adults. Boxplots for children (A) and adult (B) subjects. Within these boxplots, the box represents the interquartile range (IQR), with the bottom representing the first quartile (Q1, 25th percentile), and the top representing the third quartile (Q3, 75th percentile). The line within the box represents the median or central data point. Whiskers extending from the top and bottom of the boxes represent variability beyond quartiles typically spanning up to 1.5 times the IQR. Outliers are shown as circles or asterisks, with the corresponding subject ID indicated next to each outlier symbol. Outliers below Q1 minus 1.5 times the IQR or above Q3 plus 1.5 times the IQR are marked with circles. Those exceeding 3 times the IQR are indicated by asterisks. Children: *n* = 181 participants; Adults: *n* = 160 participants.

The distribution of stereoacuity measured with conventional and autostereoscopic tablet stereotests, plotted in a boxplot in Fig 6, with statistical analysis results detailed in Table 4, was not normally distributed (Shapiro-Wilk test, *P* all < 0.001). There were no significant differences between the conventional and autostereoscopic stereotests within the groups (Wilcoxon Signed-rank test, *P* all > 0.05), and significant agreement was observed between them (weighted Kappa coefficient, *κ* all > 0.80).

**Table 4:** Statistical results of the data.

Group	Test	Shapiro-Wilk test		Median (Inter-quartile range) log arcsec	Wilcoxon Signed-rank test		weighted Kappa coefficient	
		Statistic	*P*		Statistic	*P*	*κ*	*95% CI*
Child	Circle	0.700	<0.001	1.70 (1.60–2.00)	−1.743	0.081	0.897	[0.857, 0.937]
	Circle-AI	0.737	<0.001	1.70 (1.60–2.00)				
	Pacman	0.823	<0.001	2.10 (1.80–2.40)	−0.026	0.980	0.827	[0.769, 0.885]
	Pacman-AI	0.820	<0.001	2.10 (1.80–2.40)				
Adult	Circle	0.781	<0.001	1.70 (1.60–2.00)	−0.298	0.766	0.929	[0.906, 0.952]
	Circle-AI	0.768	<0.001	1.70 (1.60–2.11)				
	Pacman	0.725	<0.001	1.78 (1.78–2.08)	−1.567	0.117	0.852	[0.794, 0.910]
	Pacman-AI	0.682	<0.001	1.78 (1.78–2.08)				

**Fig 7:**
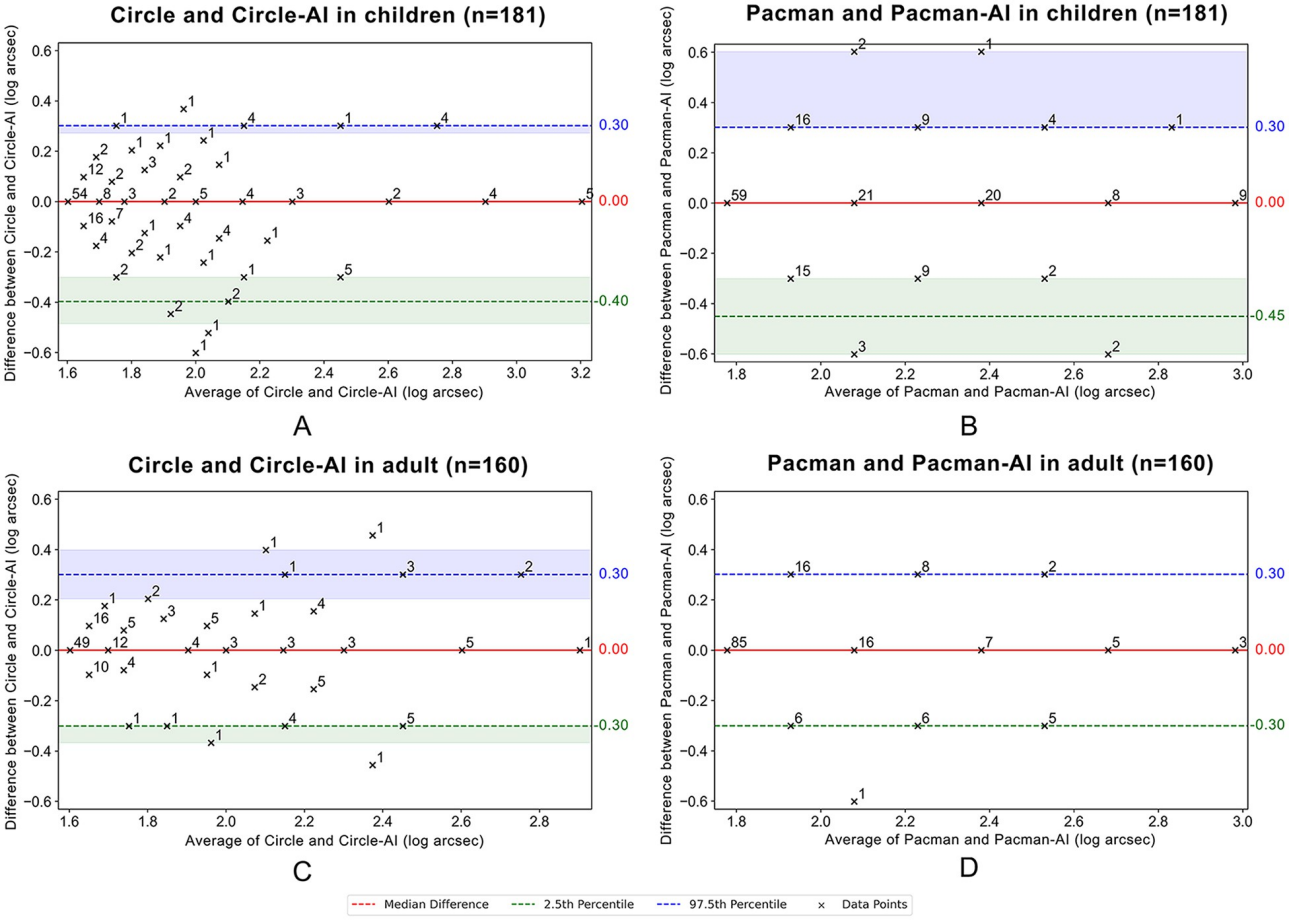
Non-parametric Bland-Altman plots between conventional and autostereoscopic 3D stereotests in both children and adults. The y-axis represents the differences between stereoacuity measurements with conventional and autostereoscopic stereotests, and the x-axis representing their average, both expressed in log arcsec. Stereoacuity thresholds measured from "Circle" and "Circle-AI" were compared in children (A) and adults (C), and those from "Pacman" and "Pacman-AI" were compared in children (B) and adults (D). Individual data points, denoted by black crosses, show the differences between the two assessments as they relate to their average, with adjacent numbers indicating the number of participants sharing the same differences. The median difference between the measurements is indicated by the red dashed line. The green dashed line signifies the 2.5th percentile of the differences, representing the lower LoA. The blue dashed line represents the 97.5th percentile, which is the upper LoA. The accompanying shaded green and blue regions represent the 95% confidence intervals for the 2.5th and 97.5th percentiles, respectively. The 95% confidence intervals were calculated using the bootstrap method, which is suitable for data without requiring assumptions about the underlying distribution. Children: *n* = 181 participants; Adults: *n* = 160 participants.

Fig 7 illustrates the non-parametric Bland-Altman plot, and the statistical results are detailed in Table 5. The median of differences was zero, indicating that the conventional and autostereoscopic stereotests gave similar results and demonstrated good agreement between the two methods. Most data points lay within the agreement limits, though a few outliers and deviations occurred. These outliers could be attributed to variability in participant responses, potentially influenced by factors such as fatigue, attention levels, familiarity with the test symbols, or differential responses to different dichoptic presentation methods.

**Table 5:** Non-parametric Bland-Altman plot for agreement between conventional and autostereoscopic 3D stereotests.

Index	Children		Adults	
	Circle vs. Circle-AI	Pacman vs. Pacman-AI	Circle vs. Circle-AI	Pacman vs. Pacman-AI
Median difference	0	0	0	0
2.5th percentile (95% CI)	−0.40 (−0.49, −0.30)	−0.45 (−0.60, −0.30)	−0.30 (−0.37, −0.30)	−0.30 (−0.31, −0.30)
97.5th percentile (95% CI)	0.30 (0.27, 0.30)	0.30 (0.30, 0.60)	0.30 (0.18, 0.40)	0.30 (0.30, 0.30)
LoA	0.70	0.75	0.60	0.60
permissible difference	0.79	1.20	0.77	0.61

All units of the data are log arcsec.

In our study, the minimal step range for the Stereo Fly Test was 10″ [31], which is the difference between 40″ and 50″, or 50″ and 60″. This minimal step range surpassed the permissible difference between the conventional and autostereoscopic stereotests (6.1″ in children and 5.8″ in adults). Similarly, the minimal step range for the TNO Stereotest was 60″, which also exceeded the permissible difference (16.0″ for children and 4.1″ for adults). This indicated that even in the most extreme condition of disagreement between the two methods, the difference was still lower than the minimum disparity increment measurable by the conventional stereotests, demonstrating the good agreement between autostereoscopic and conventional stereotests.

## Discussion

In this study, we evaluated the inter-session reliability of the AI-based autostereoscopic stereotests and their agreement with the quantitative aspects of conventional stereotests. The results showed that AI-based autostereoscopic technology performed well in stereopsis evaluation and could achieve similar outcomes using dichoptic presentation compared with anaglyphic or polarized glasses, indicating the potential effectiveness of this technology in assessing stereopsis.

In the assessment of stereopsis, the ideal scenario entails the use of corrective glasses without requiring auxiliary equipment. Several methods exist for real space evaluation, including the Frisby-Davis Distance Stereotest (FD2) for distance stereopsis [10, 32, 33] and the Frisby near stereotest for near stereopsis evaluation [10–12]. However, the widespread adoption of these methods remains limited. A notable concern is the presence of multiple depth cues during the examination [34, 35]. While certain monocular cues can be mitigated through methodological adjustments, such as employing a uniform background and ensuring a controlled light source and stable head position for the subject, some binocular cues, like vergence, are insurmountable [36]. Typically, evaluations conducted in real space yield lower stereoacuity compared to those undertaken in dichoptic presentation [37, 38]. Clinically, dichoptic presentation techniques are dominant. The TNO Stereotest, which employs anaglyphic glasses, has been a staple for over fifty years [39]. Furthermore, methods utilizing polarized glasses are even more widespread, as evidenced by tests such as the Titmus Stereotest, Randot Stereotest, Pass Test 3, Random Dot E Stereotest, Butterfly Stereo Acuity Test, Random Dot Stereo Acuity Test, Distance Randot Test, Preschool Randot Stereotest, and others [40–44].

Autostereoscopic technology is a method for dichoptic presentation that doesn’t necessitate the use of auxiliary equipment. However, as mentioned earlier, while conventional printed autostereoscopic materials can achieve dichoptic presentation, they do not allow for ensuring that each eye correctly perceives the image intended for it, thus potentially compromising the stereopsis evaluation. Numerous autostereoscopic display devices share the same limitations as printed autostereoscopic images [28, 45]. Accurate and clear 3D visualization necessitates specific distances, angles, and stable head positioning. While these conditions may be achievable in research settings or for specific subjects in controlled environments, they present challenges for routine clinical applications.

For the clinical application of autostereoscopic display technology, it’s crucial to ensure that each eye consistently receives its intended image, even if there are variations in head position or the angle of the test screen. Specifically, the right eye should always perceive the image designed for it, and not the one meant for the left eye, and vice versa. In this study, the Leia’s Lume Pad 2 was utilized to address this challenge. To our knowledge, its unique autostereoscopic display incorporating AI-based facial tracking technology. During the stereotest, all participants consistently perceived protrusion (crossed disparity) without any alternation between protrusion and indentation (uncrossed disparity). This implied that the AI-based autostereoscopic technology enables delivery of the correct images to the intended right and left eyes.

Lagstein et al. [46] evaluated the agreement between the Bernell Evaluation of Stereopsis Test (BEST) and the Randot Stereotest in measuring children’s stereopsis. The BEST employed lenticular technology, and the Randot Stereotest employed polarized glasses to achieve dichoptic presentation, which was similar to the methods used in our study. They reported that the LoA between the BEST and the Randot Stereotest ranged from -0.4816 to 0.4962 log arcsec, which was below the clinically significant threshold. In our study, the LoA between the conventional and autostereoscopic Stereo Fly test in children ranged from -0.40 to 0.30 log arcsec, while for the TNO stereotests it was -0.45 to 0.30 log arcsec. In adults, the LoA for both the conventional and autostereoscopic Stereo Fly test and the TNO stereotests ranged from -0.30 to 0.30 log arcsec. These findings show that the autostereoscopic stereotests exhibited high agreement with conventional methods. Since the AI-based autostereoscopic technology has overcome the dependence of conventional printed autostereoscopic materials on angles and stable head positioning, the high agreement between autostereoscopic and conventional stereotests indicates that this technology can achieve dichoptic presentations comparable to those provided by anaglyphic or polarized glasses. This technology provides clinicians and researchers with a novel approach for stereopsis evaluation. It offers the ability to generate a wider variety of stereograms compared to conventional stereotests. Moreover, when combined with SPR technology, it is possible to detect finer disparities.

For the autostereoscopic stereotest, in addition to ensuring accurate image delivery to the intended eyes, it is crucial to maintain a precise distance from the test page to the plane of the subject’s gaze, as this significantly influences the accuracy of the disparity cues presented. Autostereoscopic displays often face challenges in maintaining accurate depth perception when the viewer’s head or the display itself moves closer to or farther away from each other. Although our study utilized a tape measure to ensure subjects maintained the correct detection distance, this depended on the subjects’ precise cooperation, which proved challenging for children. Existing solutions, such as head-tracking and adaptive techniques, could improve viewing flexibility [47]. It would be beneficial to understand how the AI-based autostereoscopic technology integrates with these approaches in addressing viewing distance challenges during stereopsis evaluation.

Stereopsis should ideally rely solely on disparity to reflect sensory and perceptual processes [48]. However, factors such as attention, motivation, and language ability can influence stereopsis, especially in children. Nonetheless, our results showed that both children and adults perform similarly on conventional and AI-based autostereoscopic stereotests. Changes in stereoacuity greater than approximately two octaves are required to exceed the test-retest variability in most stereotests [10]. In our study, both the LoA and permissible difference of the Stereo Fly test and TNO Stereotest simulated using AI-based autostereoscopic technology were less than two octaves for both children and adults, indicating that it is a reliable method for assessing stereopsis. It is noteworthy that only the autostereoscopic TNO Stereotest for adults demonstrated moderate inter-session reliability (κ = 0.571). Similar to children, 25 adult participants had the same stereoacuity between the two measurements. However, the distribution of stereoacuity among adults was more skewed. Specifically, 24 participants in the adult group exhibited a stereoacuity of 1.8 log arcsec in both tests, compared to only 16 participants in the children’s group. This skewed stereoacuity distribution in adults increases the probability of chance agreement, resulting in reduced inter-session reliability [49]. Generally, participants with poor stereopsis tend to perform poorly on both conventional and AI-based autostereoscopic stereotests. However, significant differences in stereoacuity were observed between these methods in eight children and eight adults. Specifically, stereoacuity measured in one participant was identified as an outlier in the conventional stereotest but not in the AI-based autostereoscopic stereotest, and vice versa. This variation may be attributed to differences in test sensitivity, individual responses, and the distinct dichoptic presentation methods employed.

The Bland-Altman analysis is commonly used to assess the agreement between two different methods or to evaluate the reliability of a single method. Traditional Bland-Altman analysis compares the mean of the two methods against their difference, determining LoA based on a mean difference ± 1.96 times the standard deviation [50, 51]. However, in our study, the differences weren’t normally distributed, making the conventional Bland-Altman analysis inapplicable. Instead, we utilized the non-parametric version, which employs the median difference and sets limits based on specific percentiles, typically the 2.5th and 97.5th [52]. This approach provides a more realistic range of agreement, especially when the differences between methods exhibit skewness, outliers, or other non-normal behaviors.

The 12.4-inch tablet used in our study, despite having a resolution of 2560×1600, may not be sufficient for detailed near distance stereopsis measurements. When utilizing the integer pixel design at a distance of 0.4m, the precision of achievable disparity, which refers to the smallest detectable difference in depth, is limited to 100″. This level of detail, while acceptable for preliminary screenings, isn’t satisfactory for detailed clinical evaluations. To circumvent this drawback, we turned to SPR technology. This enhancement allowed us to finely calibrate the disparity, ensuring it matched the disparity obtained from both the TNO and the Stereo Fly test.

SPR technology uses bilinear interpolation to compute the RGB values at the edge of an object. Changes in RGB values influence the luminance perceived by the human eye, an effect that is non-linear. However, our prior research indicated that stereopsis is sufficiently tolerant within a wide range of luminance variations (30 cd/m^2^ to 240 cd/m^2^) [53]. Although the use of SPR technology may not compromise stereopsis assessments, to ensure precision, we minimized the reliance on SPR technology for generating test symbols by adjusting the test distances.

This study has several limitations: (1) The small sample size and inclusion of only participants with normal vision in this study may not represent the broader clinical population. Future research could explore the potential applications of AI-based autostereoscopic technology in measuring stereopsis and vision training for patients with strabismus or amblyopia. (2) Our study focused on the dichoptic presentation of AI-based autostereoscopic technology, simulating only commercially available stereotests rather than designing a test to detect subtle changes in disparity. (3) Our study used the Stereo Fly test and the TNO Stereotest to evaluate the inter-session reliability and agreement of AI-based autostereoscopic technology. Bringing in a broader spectrum of stereotests, such as Randot Stereotest, could further solidify the evidence supporting its potential for evaluating stereopsis. (4) Although the correct image was perceived by each eye, subtle crosstalk may not be detected.

## Conclusion

This study investigated the potential clinical applicability of AI-based autostereoscopic technology for stereopsis measurements. Capable of achieving dichoptic presentations comparable to anaglyphic or polarized glasses, this technology demonstrated high reliability and agreement with conventional stereotests. Leveraging AI-based autostereoscopic technology, grounded in face tracking, yields an optimal 3D display effect. When combined with SPR technology, it becomes viable for clinical stereopsis measurements. However, as only participants with normal vision were included in this study, future studies could investigate its application in the broader clinical population, especially in strabismus and amblyopia.

## Supporting information

S1 Data (DOCX)
