# Supplementary material for: Assessment of autostereoscopic perception using artificial intelligence-enhanced face tracking technology
Source: PLoS One. 2024 Oct 17;19(10):e0312153. doi: 10.1371/journal.pone.0312153 (PMC11486382; doi:10.1371/journal.pone.0312153)
Supplement: S1 Data — (DOCX) [file pone.0312153.s001.docx]

**Supplementary Material**

**S1 Table.**

**Inter-session reliability analysis results of the stereotest with autostereoscopic tablet in children (″)**

| **ID** | **Test 1 of Circle** | **Test 2 of Circle** | **Test 1 of Pacman** | **Test 2 of Pacman** |
| --- | --- | --- | --- | --- |
| 1 | 60 | 40 | 60 | 60 |
| 2 | 40 | 40 | 120 | 240 |
| 3 | 400 | 400 | 960 | 960 |
| 4 | 400 | 400 | 960 | 960 |
| 5 | 40 | 40 | 60 | 60 |
| 6 | 40 | 40 | 60 | 60 |
| 7 | 80 | 80 | 120 | 60 |
| 8 | 40 | 40 | 120 | 240 |
| 9 | 400 | 400 | 240 | 240 |
| 10 | 40 | 50 | 60 | 120 |
| 11 | 50 | 50 | 60 | 60 |
| 12 | 60 | 60 | 60 | 60 |
| 13 | 200 | 200 | 480 | 480 |
| 14 | 60 | 40 | 120 | 120 |
| 15 | 40 | 40 | 60 | 60 |
| 16 | 40 | 40 | 60 | 60 |
| 17 | 40 | 40 | 60 | 60 |
| 18 | 400 | 400 | 240 | 240 |
| 19 | 100 | 100 | 60 | 60 |
| 20 | 60 | 60 | 60 | 60 |
| 21 | 50 | 40 | 120 | 120 |
| 22 | 40 | 40 | 120 | 120 |
| 23 | 40 | 40 | 60 | 60 |
| 24 | 140 | 140 | 60 | 60 |
| 25 | 400 | 200 | 120 | 60 |
| 26 | 40 | 40 | 60 | 60 |
| 27 | 60 | 100 | 60 | 60 |
| 28 | 50 | 40 | 60 | 60 |
| 29 | 400 | 400 | 960 | 960 |
| 30 | 60 | 50 | 60 | 60 |

**S2 Table.**

**Inter-session reliability analysis results of the stereotest with autostereoscopic tablet in adult (″)**

| **ID** | **Test 1 of Circle** | **Test 2 of Circle** | **Test 1 of Pacman** | **Test 2 of Pacman** |
| --- | --- | --- | --- | --- |
| 1 | 40 | 40 | 60 | 60 |
| 2 | 50 | 40 | 60 | 60 |
| 3 | 50 | 50 | 60 | 60 |
| 4 | 40 | 40 | 60 | 60 |
| 5 | 40 | 40 | 120 | 60 |
| 6 | 40 | 40 | 60 | 60 |
| 7 | 40 | 40 | 60 | 60 |
| 8 | 40 | 40 | 60 | 60 |
| 9 | 40 | 40 | 60 | 60 |
| 10 | 40 | 40 | 60 | 60 |
| 11 | 40 | 40 | 60 | 60 |
| 12 | 40 | 40 | 60 | 60 |
| 13 | 40 | 40 | 60 | 60 |
| 14 | 40 | 40 | 60 | 60 |
| 15 | 50 | 40 | 60 | 60 |
| 16 | 40 | 40 | 60 | 60 |
| 17 | 40 | 40 | 60 | 60 |
| 18 | 40 | 40 | 60 | 60 |
| 19 | 40 | 40 | 60 | 60 |
| 20 | 60 | 60 | 60 | 60 |
| 21 | 40 | 40 | 60 | 60 |
| 22 | 40 | 40 | 60 | 120 |
| 23 | 50 | 50 | 60 | 60 |
| 24 | 80 | 80 | 60 | 120 |
| 25 | 80 | 80 | 120 | 60 |
| 26 | 80 | 100 | 120 | 60 |
| 27 | 40 | 40 | 60 | 60 |
| 28 | 40 | 40 | 60 | 60 |
| 29 | 60 | 40 | 60 | 60 |
| 30 | 40 | 40 | 960 | 960 |

**S3 Table.**

**Agreement analysis results of the stereotest with autostereoscopic tablet in children (″)**

| **ID** | **Circle** | **Circle-AI** | **Pacman** | **Pacman-AI** |
| --- | --- | --- | --- | --- |
| 1 | 100 | 140 | 240 | 240 |
| 2 | 200 | 400 | 480 | 240 |
| 3 | 800 | 800 | 480 | 480 |
| 4 | 50 | 50 | 120 | 240 |
| 5 | 50 | 50 | 60 | 120 |
| 6 | 50 | 40 | 60 | 60 |
| 7 | 40 | 50 | 60 | 60 |
| 8 | 100 | 80 | 120 | 120 |
| 9 | 100 | 140 | 240 | 960 |
| 10 | 40 | 60 | 60 | 120 |
| 11 | 40 | 40 | 60 | 60 |
| 12 | 60 | 80 | 120 | 240 |
| 13 | 40 | 40 | 60 | 120 |
| 14 | 80 | 200 | 60 | 240 |
| 15 | 200 | 200 | 240 | 240 |
| 16 | 50 | 50 | 120 | 120 |
| 17 | 50 | 80 | 120 | 240 |
| 18 | 80 | 80 | 240 | 240 |
| 19 | 40 | 50 | 60 | 60 |
| 20 | 50 | 50 | 60 | 120 |
| 21 | 1600 | 1600 | 960 | 960 |
| 22 | 40 | 40 | 60 | 60 |
| 23 | 40 | 60 | 240 | 60 |
| 24 | 40 | 40 | 60 | 240 |
| 25 | 400 | 400 | 240 | 240 |
| 26 | 80 | 200 | 120 | 240 |
| 27 | 40 | 40 | 60 | 60 |
| 28 | 50 | 60 | 60 | 60 |
| 29 | 40 | 60 | 120 | 120 |
| 30 | 40 | 50 | 120 | 60 |
| 31 | 40 | 50 | 240 | 480 |
| 32 | 50 | 140 | 240 | 240 |
| 33 | 100 | 200 | 240 | 240 |
| 34 | 80 | 140 | 120 | 240 |
| 35 | 50 | 60 | 120 | 240 |
| 36 | 40 | 60 | 60 | 120 |

| **ID** | **Circle** | **Circle-AI** | **Pacman** | **Pacman-AI** |
| --- | --- | --- | --- | --- |
| 37 | 50 | 200 | 120 | 120 |
| 38 | 80 | 100 | 120 | 120 |
| 39 | 40 | 80 | 120 | 120 |
| 40 | 100 | 140 | 480 | 240 |
| 41 | 50 | 60 | 60 | 120 |
| 42 | 200 | 400 | 240 | 240 |
| 43 | 80 | 100 | 240 | 240 |
| 44 | 50 | 60 | 240 | 240 |
| 45 | 40 | 40 | 60 | 60 |
| 46 | 200 | 100 | 120 | 60 |
| 47 | 80 | 80 | 120 | 60 |
| 48 | 80 | 40 | 120 | 120 |
| 49 | 40 | 40 | 60 | 60 |
| 50 | 140 | 60 | 240 | 60 |
| 51 | 50 | 60 | 60 | 60 |
| 52 | 200 | 200 | 480 | 120 |
| 53 | 40 | 40 | 60 | 120 |
| 54 | 800 | 800 | 480 | 480 |
| 55 | 40 | 50 | 240 | 120 |
| 56 | 40 | 40 | 60 | 60 |
| 57 | 100 | 140 | 240 | 120 |
| 58 | 50 | 40 | 120 | 60 |
| 59 | 200 | 100 | 120 | 240 |
| 60 | 50 | 40 | 120 | 60 |
| 61 | 80 | 100 | 240 | 240 |
| 62 | 50 | 50 | 120 | 60 |
| 63 | 100 | 100 | 240 | 240 |
| 64 | 140 | 100 | 240 | 240 |
| 65 | 50 | 40 | 120 | 120 |
| 66 | 80 | 60 | 240 | 240 |
| 67 | 200 | 100 | 60 | 60 |
| 68 | 100 | 100 | 240 | 240 |
| 69 | 60 | 40 | 60 | 120 |
| 70 | 140 | 200 | 60 | 120 |
| 71 | 100 | 100 | 240 | 120 |
| 72 | 40 | 40 | 240 | 240 |
| 73 | 50 | 140 | 240 | 120 |
| 74 | 40 | 40 | 60 | 60 |
| 75 | 200 | 200 | 240 | 120 |
| 76 | 40 | 40 | 60 | 60 |
| 77 | 50 | 50 | 120 | 60 |
| 78 | 40 | 50 | 60 | 60 |

| **ID** | **Circle** | **Circle-AI** | **Pacman** | **Pacman-AI** |
| --- | --- | --- | --- | --- |
| 79 | 40 | 40 | 60 | 60 |
| 80 | 140 | 140 | 120 | 120 |
| 81 | 200 | 100 | 240 | 240 |
| 82 | 50 | 80 | 240 | 120 |
| 83 | 40 | 50 | 60 | 60 |
| 84 | 50 | 40 | 60 | 120 |
| 85 | 40 | 40 | 60 | 60 |
| 86 | 80 | 60 | 120 | 120 |
| 87 | 40 | 40 | 120 | 120 |
| 88 | 60 | 40 | 120 | 120 |
| 89 | 140 | 140 | 240 | 120 |
| 90 | 40 | 50 | 120 | 120 |
| 91 | 40 | 40 | 60 | 60 |
| 92 | 60 | 50 | 120 | 120 |
| 93 | 200 | 400 | 480 | 480 |
| 94 | 800 | 800 | 960 | 960 |
| 95 | 80 | 50 | 60 | 60 |
| 96 | 50 | 40 | 60 | 60 |
| 97 | 40 | 50 | 60 | 120 |
| 98 | 40 | 40 | 60 | 60 |
| 99 | 40 | 40 | 60 | 60 |
| 100 | 140 | 140 | 480 | 480 |
| 101 | 40 | 40 | 60 | 60 |
| 102 | 40 | 40 | 60 | 60 |
| 103 | 40 | 40 | 60 | 60 |
| 104 | 50 | 40 | 120 | 120 |
| 105 | 60 | 60 | 60 | 120 |
| 106 | 50 | 50 | 60 | 120 |
| 107 | 1600 | 1600 | 960 | 960 |
| 108 | 40 | 40 | 120 | 60 |
| 109 | 40 | 40 | 120 | 60 |
| 110 | 40 | 40 | 60 | 60 |
| 111 | 50 | 40 | 60 | 60 |
| 112 | 40 | 40 | 60 | 60 |
| 113 | 40 | 40 | 60 | 60 |
| 114 | 200 | 400 | 240 | 240 |
| 115 | 140 | 140 | 240 | 480 |
| 116 | 40 | 40 | 60 | 60 |
| 117 | 40 | 40 | 60 | 60 |
| 118 | 40 | 40 | 120 | 60 |
| 119 | 40 | 40 | 60 | 60 |
| 120 | 50 | 40 | 60 | 60 |

| **ID** | **Circle** | **Circle-AI** | **Pacman** | **Pacman-AI** |
| --- | --- | --- | --- | --- |
| 121 | 60 | 60 | 60 | 60 |
| 122 | 40 | 40 | 60 | 60 |
| 123 | 50 | 50 | 120 | 60 |
| 124 | 50 | 60 | 60 | 60 |
| 125 | 60 | 60 | 480 | 240 |
| 126 | 40 | 40 | 60 | 60 |
| 127 | 40 | 40 | 60 | 60 |
| 128 | 40 | 50 | 60 | 60 |
| 129 | 1600 | 1600 | 960 | 960 |
| 130 | 50 | 60 | 120 | 120 |
| 131 | 60 | 50 | 240 | 240 |
| 132 | 140 | 80 | 960 | 480 |
| 133 | 200 | 400 | 120 | 120 |
| 134 | 40 | 40 | 120 | 60 |
| 135 | 40 | 40 | 60 | 60 |
| 136 | 40 | 40 | 120 | 60 |
| 137 | 40 | 40 | 240 | 120 |
| 138 | 50 | 40 | 240 | 120 |
| 139 | 40 | 40 | 120 | 120 |
| 140 | 40 | 50 | 60 | 60 |
| 141 | 40 | 50 | 60 | 60 |
| 142 | 40 | 40 | 60 | 60 |
| 143 | 100 | 60 | 240 | 240 |
| 144 | 40 | 40 | 60 | 60 |
| 145 | 40 | 50 | 60 | 120 |
| 146 | 40 | 40 | 60 | 60 |
| 147 | 40 | 40 | 120 | 120 |
| 148 | 1600 | 1600 | 960 | 960 |
| 149 | 100 | 100 | 120 | 60 |
| 150 | 60 | 100 | 240 | 240 |
| 151 | 800 | 400 | 480 | 240 |
| 152 | 1600 | 1600 | 960 | 960 |
| 153 | 40 | 40 | 60 | 120 |
| 154 | 50 | 40 | 60 | 60 |
| 155 | 40 | 40 | 60 | 60 |
| 156 | 40 | 40 | 60 | 60 |
| 157 | 40 | 40 | 60 | 60 |
| 158 | 400 | 200 | 480 | 480 |
| 159 | 100 | 100 | 480 | 480 |
| 160 | 40 | 50 | 60 | 60 |
| 161 | 40 | 40 | 60 | 60 |
| 162 | 40 | 40 | 60 | 60 |

| **ID** | **Circle** | **Circle-AI** | **Pacman** | **Pacman-AI** |
| --- | --- | --- | --- | --- |
| 163 | 400 | 400 | 960 | 960 |
| 164 | 60 | 200 | 120 | 120 |
| 165 | 80 | 100 | 120 | 240 |
| 166 | 40 | 40 | 60 | 60 |
| 167 | 40 | 40 | 120 | 60 |
| 168 | 800 | 400 | 960 | 960 |
| 169 | 40 | 50 | 120 | 240 |
| 170 | 800 | 400 | 480 | 480 |
| 171 | 50 | 40 | 120 | 120 |
| 172 | 40 | 80 | 120 | 60 |
| 173 | 80 | 60 | 60 | 240 |
| 174 | 800 | 800 | 480 | 480 |
| 175 | 40 | 40 | 60 | 60 |
| 176 | 100 | 80 | 240 | 960 |
| 177 | 40 | 40 | 60 | 60 |
| 178 | 800 | 400 | 960 | 960 |
| 179 | 40 | 40 | 60 | 60 |
| 180 | 40 | 40 | 60 | 60 |
| 181 | 40 | 50 | 60 | 60 |

**S4 Table.**

**Agreement analysis results of the stereotest with autostereoscopic tablet in adult (″)**

| **ID** | **Circle** | **Circle-AI** | **Pacman** | **Pacman-AI** |
| --- | --- | --- | --- | --- |
| 1 | 40 | 40 | 60 | 60 |
| 2 | 100 | 140 | 120 | 120 |
| 3 | 80 | 50 | 120 | 120 |
| 4 | 40 | 40 | 60 | 60 |
| 5 | 40 | 40 | 60 | 60 |
| 6 | 40 | 40 | 60 | 60 |
| 7 | 40 | 40 | 60 | 60 |
| 8 | 50 | 40 | 60 | 60 |
| 9 | 50 | 50 | 60 | 60 |
| 10 | 40 | 40 | 60 | 60 |
| 11 | 50 | 40 | 60 | 60 |
| 12 | 50 | 50 | 60 | 60 |
| 13 | 40 | 40 | 120 | 60 |
| 14 | 200 | 400 | 120 | 60 |
| 15 | 100 | 100 | 60 | 120 |
| 16 | 80 | 60 | 120 | 120 |
| 17 | 200 | 200 | 240 | 240 |
| 18 | 140 | 200 | 120 | 120 |
| 19 | 50 | 40 | 60 | 60 |
| 20 | 50 | 50 | 120 | 60 |
| 21 | 40 | 40 | 60 | 60 |
| 22 | 40 | 50 | 60 | 60 |
| 23 | 60 | 50 | 240 | 120 |
| 24 | 140 | 200 | 240 | 240 |
| 25 | 40 | 40 | 60 | 60 |
| 26 | 50 | 60 | 120 | 60 |
| 27 | 140 | 200 | 120 | 240 |
| 28 | 60 | 50 | 120 | 60 |
| 29 | 100 | 200 | 120 | 120 |
| 30 | 80 | 60 | 60 | 60 |
| 31 | 200 | 400 | 120 | 60 |
| 32 | 40 | 40 | 60 | 60 |
| 33 | 50 | 40 | 60 | 60 |
| 34 | 50 | 50 | 60 | 60 |
| 35 | 40 | 40 | 60 | 60 |
| 36 | 50 | 40 | 120 | 60 |

| **ID** | **Circle** | **Circle-AI** | **Pacman** | **Pacman-AI** |
| --- | --- | --- | --- | --- |
| 37 | 400 | 400 | 240 | 480 |
| 38 | 200 | 140 | 240 | 120 |
| 39 | 140 | 140 | 120 | 120 |
| 40 | 60 | 50 | 60 | 60 |
| 41 | 80 | 80 | 120 | 60 |
| 42 | 40 | 50 | 60 | 60 |
| 43 | 40 | 40 | 60 | 60 |
| 44 | 50 | 50 | 60 | 60 |
| 45 | 50 | 50 | 60 | 60 |
| 46 | 400 | 200 | 240 | 480 |
| 47 | 100 | 80 | 120 | 60 |
| 48 | 60 | 50 | 60 | 60 |
| 49 | 40 | 50 | 60 | 60 |
| 50 | 40 | 40 | 60 | 60 |
| 51 | 40 | 40 | 60 | 60 |
| 52 | 200 | 200 | 240 | 240 |
| 53 | 50 | 50 | 60 | 60 |
| 54 | 40 | 50 | 60 | 120 |
| 55 | 800 | 400 | 960 | 960 |
| 56 | 80 | 100 | 60 | 120 |
| 57 | 60 | 50 | 60 | 60 |
| 58 | 400 | 400 | 240 | 480 |
| 59 | 140 | 140 | 120 | 120 |
| 60 | 40 | 40 | 60 | 60 |
| 61 | 40 | 40 | 60 | 60 |
| 62 | 50 | 40 | 120 | 60 |
| 63 | 40 | 40 | 60 | 60 |
| 64 | 100 | 80 | 120 | 240 |
| 65 | 200 | 80 | 120 | 240 |
| 66 | 100 | 200 | 240 | 120 |
| 67 | 40 | 40 | 60 | 60 |
| 68 | 100 | 80 | 120 | 60 |
| 69 | 50 | 60 | 60 | 60 |
| 70 | 40 | 40 | 60 | 60 |
| 71 | 40 | 50 | 60 | 60 |
| 72 | 40 | 40 | 60 | 60 |
| 73 | 200 | 140 | 120 | 240 |
| 74 | 80 | 60 | 120 | 60 |
| 75 | 50 | 50 | 60 | 60 |
| 76 | 200 | 400 | 240 | 480 |
| 77 | 40 | 40 | 60 | 60 |
| 78 | 50 | 40 | 60 | 60 |

| **ID** | **Circle** | **Circle-AI** | **Pacman** | **Pacman-AI** |
| --- | --- | --- | --- | --- |
| 79 | 100 | 200 | 240 | 120 |
| 80 | 50 | 100 | 120 | 240 |
| 81 | 40 | 50 | 60 | 60 |
| 82 | 50 | 40 | 120 | 120 |
| 83 | 80 | 80 | 240 | 120 |
| 84 | 40 | 40 | 60 | 60 |
| 85 | 40 | 40 | 60 | 60 |
| 86 | 100 | 200 | 60 | 120 |
| 87 | 50 | 40 | 60 | 60 |
| 88 | 40 | 40 | 60 | 60 |
| 89 | 400 | 400 | 480 | 480 |
| 90 | 200 | 400 | 480 | 240 |
| 91 | 40 | 40 | 60 | 60 |
| 92 | 40 | 40 | 60 | 60 |
| 93 | 200 | 100 | 240 | 240 |
| 94 | 40 | 40 | 60 | 120 |
| 95 | 40 | 40 | 60 | 60 |
| 96 | 200 | 140 | 120 | 120 |
| 97 | 400 | 400 | 480 | 480 |
| 98 | 400 | 140 | 240 | 120 |
| 99 | 140 | 200 | 240 | 240 |
| 100 | 80 | 80 | 120 | 120 |
| 101 | 40 | 50 | 60 | 60 |
| 102 | 60 | 140 | 120 | 120 |
| 103 | 40 | 40 | 60 | 60 |
| 104 | 40 | 40 | 60 | 60 |
| 105 | 50 | 40 | 60 | 60 |
| 106 | 50 | 40 | 120 | 60 |
| 107 | 40 | 40 | 60 | 60 |
| 108 | 50 | 50 | 60 | 60 |
| 109 | 40 | 40 | 60 | 60 |
| 110 | 50 | 50 | 60 | 60 |
| 111 | 50 | 50 | 60 | 120 |
| 112 | 40 | 40 | 60 | 60 |
| 113 | 50 | 60 | 60 | 60 |
| 114 | 140 | 200 | 120 | 240 |
| 115 | 50 | 40 | 60 | 60 |
| 116 | 40 | 40 | 60 | 60 |
| 117 | 40 | 40 | 60 | 60 |
| 118 | 200 | 140 | 120 | 120 |
| 119 | 100 | 100 | 120 | 120 |
| 120 | 800 | 800 | 960 | 960 |

| **ID** | **Circle** | **Circle-AI** | **Pacman** | **Pacman-AI** |
| --- | --- | --- | --- | --- |
| 121 | 100 | 80 | 120 | 60 |
| 122 | 80 | 50 | 120 | 120 |
| 123 | 40 | 40 | 60 | 60 |
| 124 | 40 | 40 | 60 | 60 |
| 125 | 140 | 140 | 240 | 120 |
| 126 | 800 | 400 | 480 | 480 |
| 127 | 40 | 40 | 60 | 60 |
| 128 | 100 | 100 | 120 | 60 |
| 129 | 40 | 50 | 60 | 60 |
| 130 | 50 | 40 | 60 | 60 |
| 131 | 40 | 40 | 60 | 60 |
| 132 | 50 | 60 | 60 | 60 |
| 133 | 400 | 200 | 240 | 240 |
| 134 | 200 | 200 | 240 | 120 |
| 135 | 50 | 40 | 60 | 60 |
| 136 | 40 | 80 | 60 | 60 |
| 137 | 60 | 40 | 60 | 60 |
| 138 | 40 | 40 | 60 | 60 |
| 139 | 40 | 40 | 60 | 240 |
| 140 | 80 | 80 | 120 | 120 |
| 141 | 40 | 50 | 60 | 60 |
| 142 | 40 | 40 | 60 | 60 |
| 143 | 50 | 40 | 60 | 60 |
| 144 | 40 | 40 | 60 | 60 |
| 145 | 50 | 50 | 120 | 120 |
| 146 | 40 | 40 | 60 | 60 |
| 147 | 140 | 100 | 480 | 240 |
| 148 | 50 | 40 | 60 | 60 |
| 149 | 40 | 40 | 60 | 60 |
| 150 | 100 | 140 | 240 | 240 |
| 151 | 40 | 40 | 60 | 60 |
| 152 | 40 | 50 | 60 | 60 |
| 153 | 40 | 40 | 60 | 60 |
| 154 | 200 | 400 | 480 | 480 |
| 155 | 40 | 40 | 60 | 60 |
| 156 | 400 | 200 | 480 | 480 |
| 157 | 140 | 400 | 960 | 960 |
| 158 | 400 | 400 | 240 | 480 |
| 159 | 100 | 80 | 120 | 60 |
| 160 | 40 | 40 | 60 | 60 |
